# Autophagy in Cisplatin Nephrotoxicity during Cancer Therapy

**DOI:** 10.3390/cancers13225618

**Published:** 2021-11-10

**Authors:** Xiaoru Hu, Zhengwei Ma, Lu Wen, Siyao Li, Zheng Dong

**Affiliations:** 1 Hunan Key Laboratory of Kidney Disease and Blood Purification, Department of Nephrology, The Second Xiangya Hospital of Central South University, Changsha 410011, China; 188201029@csu.edu.cn (X.H.); 188201019@csu.edu.cn (L.W.); lisiyao0716@csu.edu.cn (S.L.); 2 Department of Cellular Biology and Anatomy, Medical College of Georgia, Augusta University, Augusta, GA 30912, USA; ZMA@augusta.edu; 3 Charlie Norwood VA Medical Center, Augusta, GA 30912, USA

**Keywords:** cisplatin, autophagy, acute kidney injury, chronic kidney disease, nephrotoxicity, cancer therapy

## Abstract

**Simple Summary:**

Cisplatin is a broadly used chemotherapy drug, but its use and efficacy are limited by its nephrotoxicity. Autophagy protects against kidney injury during cisplatin exposure but may reduce the efficacy of chemotherapy by protecting cancer cells. In this review, we describe the role and regulation of autophagy in cisplatin-induced nephrotoxicity and discuss the therapeutic advances and challenges of targeting autophagy in chemotherapy.

**Abstract:**

Cisplatin is a widely used chemotherapeutic agent but its clinical use is often limited by nephrotoxicity. Autophagy is a lysosomal degradation pathway that removes protein aggregates and damaged or dysfunctional cellular organelles for maintaining cell homeostasis. Upon cisplatin exposure, autophagy is rapidly activated in renal tubule cells to protect against acute cisplatin nephrotoxicity. Mechanistically, the protective effect is mainly related to the clearance of damaged mitochondria via mitophagy. The role and regulation of autophagy in chronic kidney problems after cisplatin treatment are currently unclear, despite the significance of research in this area. In cancers, autophagy may prevent tumorigenesis, but autophagy may reduce the efficacy of chemotherapy by protecting cancer cells. Future research should focus on developing drugs that enhance the anti-tumor effects of cisplatin while protecting kidneys during cisplatin chemotherapy.

## 1. Introduction

Cisplatin is a potent chemotherapy drug used for the treatment of various types of tumors [1], but it has remarkable side effects or toxicity in normal tissues [2]. The kidney is highly vulnerable to cisplatin toxicity due to the accumulation of cisplatin in renal tubule cells [3]. Acute kidney injury occurs in 20–30% of patients and manifests as kidney cell death, tissue damage, rapid loss of renal function or renal failure, and even death [4]. Following cisplatin chemotherapy, a significant portion of cancer patients develop chronic kidney problems [5]. Therefore, it is vital to explore the mechanism of the nephrotoxicity of cisplatin and identify preventive or protective measures.

Autophagy is a lysosomal degradation pathway that clears dysfunctional or obsolete cytoplasmic components [6,7]. In the kidney, the basal level of autophagy plays a role in maintaining renal cell homeostasis and function under normal physiological conditions. Autophagy is induced in response to cellular stress when the kidney is diseased or exposed to insults or toxins, such as cisplatin. Autophagy is generally considered pivotal in promoting cell survival and protecting against acute cisplatin nephrotoxicity [8,9,10,11]. Autophagy also participates in the regulation of maladaptive kidney repair and renal fibrosis after acute kidney injury and during the progression of chronic kidney disease [12,13,14,15,16,17]. However, little is known about the role and regulation of autophagy in the development of chronic kidney problems after cisplatin exposure.

In cancers, the role of autophagy is Janus-faced. On the one hand, it may limit or prevent tumorigenesis, but, on the other hand, autophagy may reduce the efficacy of cancer therapy by promoting cancer cell survival [18]. Autophagy induction by cisplatin is associated with the development of cisplatin resistance in many types of cancer cells, including bladder cancer, esophageal cancer [19], lung cancer [20], ovarian cancer [21], and bone cancer [22]. Moreover, the association between the loss of autophagy genes and the decrease in other tumors’ sensitivity to chemotherapy drugs further highlights the significance of autophagy in cancer suppression in some tumors [23]. Therefore, autophagy upregulation can protect the kidneys against acute cisplatin injury, but its effect on cancers is context-dependent. When targeting autophagy as a strategy for kidney protection, the effect on tumors must be considered.

This review analyzes the current research about autophagy in cisplatin-induced acute and chronic kidney diseases from its occurrence to function and regulation. We further discuss the therapeutic potential and the challenges of targeting autophagy for kidney protection in cisplatin-mediated chemotherapy.

## 2. Cisplatin Nephrotoxicity

Cisplatin is efficacious for solid tumor treatment, either alone or in combination with other therapies, but it also causes toxicity in multiple organs and tissues, especially in the kidney [1,24]. Approximately 20–30% of patients who receive cisplatin develop acute kidney injury (AKI), which is characterized by rapid loss of renal function, the accumulation of end products of nitrogen metabolism, and the disturbance of water and electrolytes [25]. The long-term effects of cisplatin on the kidney are not entirely understood. Brillet et al. [26] and Latcha et al. [5] reported that cisplatin might lead to a subclinical but persistent low glomerular filtration rate. Therefore, a thorough understanding of AKI and CKD induced by cisplatin is necessary for clinical treatment.

### 2.1. Cisplatin-Induced Acute Kidney Injury

Cisplatin nephrotoxicity is a multifactorial process including various pathophysiological events, such as microvascular disorders, tubular injury, tubular cell death, and inflammatory response. Among them, tubular cell injury and death is a critical pathological feature [27,28,29]. In experimental models of AKI, the proximal tubule, especially the S3 segment, is highly sensitive and most vulnerable to damage [30,31]. The renal tubule damages caused by cisplatin are often manifested as tubule dilation, cast formation, and tubular cell apoptosis and necrosis [32,33].

Many studies have focused on the cellular and molecular mechanisms of tubular damage, especially proximal tubular injury. Renal tubular cells mainly uptake cisplatin through organic cation transporters 2 (OCT2) [34] and copper transporter 1 (CTR1) [35]. After entry into the cell, cisplatin may undergo a series of bio-activation processes to generate various toxic metabolites, which are catalyzed by γ-glutamyl transpeptidase (GGT) and cysteine-S-conjugate β-lyase [36]. Then, it may bind to DNA, leading to inter- and intrastrand cross-links [37], causing DNA damage and DNA-damage response [38,39], resulting in cell cycle arrest and cell death [40]. Multiple signaling pathways are activated upon cisplatin exposure, such as mitogen-activated protein kinases (MAPKs) [41] and the p53-DNA damage response pathway [42,43], leading to apoptosis, necroptosis [44], and ferroptosis [45]. Cisplatin also evokes oxidative stress [46], ER stress [47], mitochondrial dysfunction [48].

### 2.2. Cisplatin-Induced Chronic Effects in the Kidney

Currently, a common regimen of cisplatin treatment is weekly administration of relatively low doses of cisplatin for several cycles. While this regimen reduces the side effects of cisplatin, a significant portion of patients still develop acute kidney injury, and some progress into chronic kidney disease. Repeated low-dose cisplatin (RLDC) treatment models were established to study chronic kidney problems after cisplatin treatment.

In 2011, we reported the first RLDC model, in which tumor-bearing mice were treated weekly with 10 mg/kg cisplatin for 4 weeks to study the effects in tumors and kidneys [49]. Subsequently, several mouse models of RLDC treatment were established with different animal ages, animal strains, the dosage of cisplatin, and observation time [50,51,52,53,54,55]. The aging FVB/n mice were more resistant to 7 mg/kg cisplatin weekly for 4 weeks than young mice, which showed less inflammation and fibrosis [56]. Moreover, C57BL/6 mice require a higher dose of cisplatin to induce renal fibrosis than FVB/n mice [57]. In addition to age and strain, dosing regimens influence fibrotic changes; 10 mg/kg cisplatin once a week for three times and 7 mg/kg once a week for 4 weeks induced moderate renal interstitial fibrosis in male FVB/N mice [55,58]. C57BL/6 mice treated with 8 mg/kg cisplatin weekly for 4 weeks [51] and FVB/n mice treated with 7 mg/kg cisplatin weekly for 4 weeks [55,59] both showed a time-dependent increase in renal fibrosis.

Although renal fibrosis is a hallmark of progressive kidney disease, there is evidence that fibrosis may not be critical to CKD development after RLDC treatment. Landau et al. [50] emphasized that fibrosis may not play a prominent role in CKD progression in C57BL/6 mice after two doses of 15 mg/kg cisplatin with an interval of two weeks. Consistently, this model only had a minor increase in renal fibrosis, despite a significant loss of renal function [53], as did the mice receiving three weekly injections of 9 mg/kg cisplatin [52]. Thus, in these relatively severe injury models induced by relatively high doses of cisplatin, unresolved tubular damage and capillary rarefaction, rather fibrosis, are closely associated with CKD progression [50,58].

In addition to renal fibrosis, there are still other pathogenesis studied in this model. Macrophage activation [51,60] with the secretion of inflammatory factors [51,54,58,59], such as *Tnfα*, *Csf1*, *Mcp1*, and *Cxcl1*, occurred in kidneys after RLDC treatment as well. Interestingly, several studies [50,51,53] showed atubular glomeruli in cisplatin-induced CKD, associated with renal function decline. Growing evidence suggests that tubular injury affects glomerular pathophysiology, potentially due to impaired tubuloglomerular cross-talk, peritubular inflammation, or PTC rarefaction. Thus, the glomerular injury may occur after tubular injury [58]. Finally, antioxidant [60] and anti-senescence compounds [61,62] partially delayed CKD progression after RLDC treatment, suggesting the involvement of reactive oxygen species and cell senescence in the pathogenesis.

Collectively, RLDC-induced pathological changes include tubular atrophy, tubulointerstitial fibrosis, chronic inflammatory infiltration, capillary dysfunction, tubular cell death, notable atubular glomeruli, and glomerulosclerosis in some circumstances [59], leading to kidney atrophy and a decline in kidney function. The occurrence of these pathological features may depend on the experimental models, especially the severity of initial kidney injury.

## 3. Autophagy in Cisplatin-Induced AKI

### 3.1. Basics of Autophagy

#### 3.1.1. Three Forms of Autophagy

**Figure 1:**
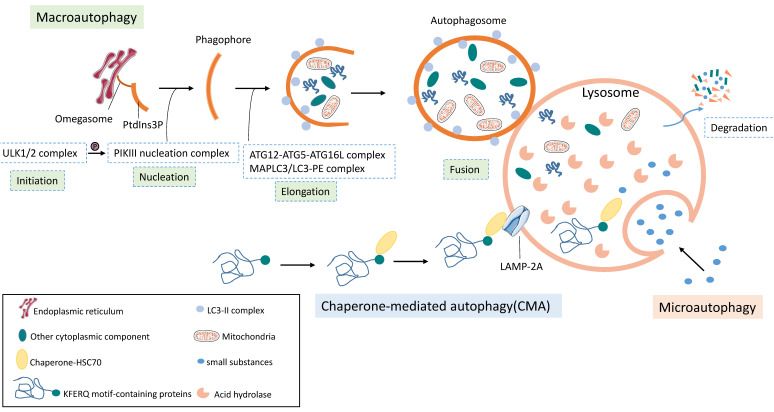
The process of autophagy. According to the type of cargo and delivery method, autophagy can be classified as macroautophagy, microautophagy, and chaperone-mediated autophagy (CMA). Macroautophagy is a process that autophagosome fuses with a lysosome to form an autolysosome, which mainly degrades dysfunctional or obsolete cytoplasmic components. Several complexes composed of autophagy-related proteins (ATG) act synergistically with membrane transport components resulting in the formation of autophagosome. CMA is a highly selective pathway, KFERQ motif-containing proteins recognized by Chaperone-HSC70 binds to LAMP-2A located in lysosome to be degraded. Small cytoplasmic substances are engulfed directly by the lysosome, called microautophagy.

Autophagy is a highly conserved cellular process of catabolism that degrades cytoplasmic components. Depending on the type of cargo and delivery method, autophagy can be classified as macroautophagy, microautophagy, and chaperone mediated autophagy. Macroautophagy (hereafter termed autophagy) is a “bulk” degradation of large cytoplasmic materials that are engulfed by autophagosomes, which fuse with lysosomes for degradation [63]. Microautophagy involves the direct sequestration of relatively small cytoplasmic substances into lysosomes through invagination of the lysosomal membrane [64]. Chaperone mediated autophagy (CMA) is a highly selective pathway in which chaperone-HSC70 recognizes the KFERQ motif-containing proteins and targets the substrate to the lysosome surface. Then, this substrate protein-chaperone complex binds to lysosome-associated membrane protein type 2A (LAMP-2A) to access the lysosome for degradation [65] (Figure 1).

#### 3.1.2. Overview of Autophagy

Autophagy is a dynamic and complex process involving a series of cellular events. The core events are the formation of autophagosomes and autolysosomes. Several complexes composed of autophagy-related proteins (ATG) act synergistically with membrane transport components resulting in the formation of the autophagosome. The entire process of autophagy includes five distinct stages: initiation, vesicle nucleation, vesicle elongation, vesicle fusion, and cargo degradation. Initially, one cell compartment consisting of lipid bilayer membranes enriched for phosphatidylinositol 3-phosphate (PtdIns3P), called omegasome, forms on the endoplasmic reticulum (ER) membrane [66]. Meanwhile, the ULK1/2 complex senses the stress signal to activate the PIKIII nucleation complex by phosphorylating its component Beclin-1 [67], thus promoting vesicle nucleation and elongation to cause phagophores (also called “isolation membranes”) formation on the site of the omegasome. Then, two ubiquitin (Ub)-like conjugation systems, the ATG12-ATG5-ATG16L complex and the microtubule-associated protein light chain 3-phosphatidyl ethanolamine (MAPLC3/LC3-PE), help the phagophore expand to form autophagosome [68]. Finally, the autophagosome fuses with a lysosome to form an autolysosome, which degrades the autophagosome inner membrane and the engulfed cytoplasmic substrates by acidic lysosomal hydrolases [7] (Figure 1).

### 3.2. Induction of Autophagy in Cisplatin AKI

In 2008, two independent studies [69,70] reported the first evidence of autophagy activation in cisplatin-induced acute nephrotoxicity. While Yang et al. [69] showed autophagy activation during cisplatin treatment of LLC-PK1 cells, we [70] demonstrated autophagy induction during cisplatin treatment of rat proximal tubular cells (RPTCs) and acute nephrotoxicity in mice induced by a single dose of 30 mg/kg cisplatin. Notably, both studies indicated that blockade of autophagy enhanced apoptosis during cisplatin treatment of renal tubular cells, suggesting a protective role of autophagy.

Cisplatin causes multiple forms of cellular stress, such as DNA damage, mitochondrial damage, ROS, and ER stress, which may play a role in autophagy activation in cisplatin-induced AKI.

#### 3.2.1. Oxidative Stress and Autophagy

Oxidative stress, with a significant role in the pathogenesis of cisplatin nephrotoxicity [71], may regulate autophagy in cisplatin AKI. This is suggested by the effects of two regulators of ROS production, heme oxygenase-1 (HO-1) and NAD(P)H: quinone oxidoreductase 1 (NQO1). HO-1, a microsomal antioxidase that catalyzes the transformation of heme to biliverdin releasing iron and carbon monoxide, was reported to inhibit autophagy in cisplatin AKI [72]. In particular, HO-1-deficient mice had significantly more autophagosomes, even under control conditions. In addition, overexpression of HO-1 in cells significantly lowered the levels of reactive oxygen species, led to a delay in autophagy progression, and decreased cell death after cisplatin injury. Thus, HO-1 may suppress autophagy via its anti-oxidant activity. NQO1 is another cytoprotective gene with antioxidant function. Kim et al. [73] reported that cisplatin-induced ROS production and NQO1 expression in cisplatin AKI, together with elevated tubular injury and death. Notably, NQO1 knockdown cells and NQO1-KO mice showed more ROS production and increased expression of autophagy-associated protein than their wild-type counterparts. One possibility is that increased ROS production in NQO1-deficient cells and mice led to autophagy activation for protection and adaptation to the stress. Together, these studies imply that oxidative stress may promote autophagy induction in cisplatin AKI.

#### 3.2.2. Endoplasmic Reticulum Stress and Autophagy

ER stress is induced along with autophagy after both cisplatin exposure [74,75] and ischemia-reperfusion (IR) injury [76]. Whether autophagy induction is related to ER stress was confirmed by pharmacological approaches. For example, inhibitors of ER stress suppressed cyclosporine-induced autophagy [75] and activators of ER stress notably increased autophagy in renal proximal tubular cells [77]. Our recent study has further demonstrated a reciprocal regulation between ER stress and autophagy in renal tubular cells. Specifically, ER stress induces autophagy leading to fibrotic changes in renal tubular cells, whereas autophagy, upon activation, reduces ER stress in these cells providing a negative feedback mechanism. ER stress might affect the induction of autophagy via regulating calcium release, REDD1/mTOR, and AKT/mTOR pathways. Moreover, the autophagy machinery components are tightly regulated by unfolded protein response (UPR) [78]. Gozuacik et al. [79] showed that death-associated protein kinase1 (DAPK1) was induced by ER stress and regulated autophagy via the phosphorylation of Beclin 1, and autophagy was inhibited in *Dapk-*null cells in tunicamycin-induced kidney injury. Conceivably, moderate ER stress activates cytoprotective mechanisms such as UPR and autophagy, whereas severe ER stress leads to irreversible cell damage and cell death. Delineating the molecular basis of this shift may lead to new strategies for reducing nephrotoxicity in cisplatin chemotherapy.

#### 3.2.3. Mitochondrial Damage and Mitophagy

**Figure 2:**
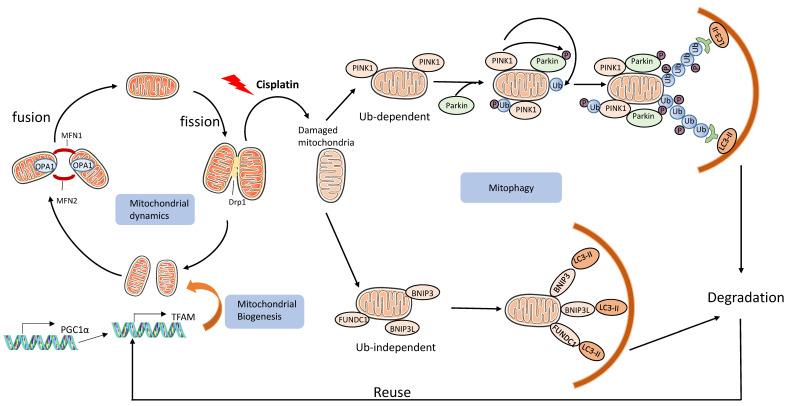
Mitochondrial quality control in cisplatin injury. Mitochondria biogenesis, dynamics, and mitophagy are important mechanisms of mitochondrial quality control. PGC-1α and TFAM are two important transcription factors for mitochondrial biogenesis. The life cycle of mitochondria begins with the growth and division, and ends with the degradation via mitophagy. In cisplatin nephrotoxicity, mitochondria are damaged and then eliminated by mitophagy.

One main event of renal tubular injury is mitochondrial damage, manifested by structural disruption and functionally by decreased cellular respiration and ATP production [33,80]. Usually, mammalian cells have multiple mechanisms for quality control, including mitochondrial biogenesis, dynamics, and mitophagy; however, these mechanisms are disrupted during cell stress or injury resulting in mitochondrial damage [81]. Mitophagy removes obsolete or damaged mitochondria to maintain mitochondrial and cellular homeostasis [8,82] (Figure 2).

Mitochondria are dynamic organelles that sustain their shape or morphology via two opposing processes, fission mediated by Drp1 and fusion regulated by mitofusin 1 (MFN1), MFN2, and OPA1 [83]. Mitophagy is a form of selective autophagy whereby dysfunctional mitochondria are degraded via autophagy. Mitochondrial dynamics and mitophagy are intimately related to each other. In 2009, our group reported that mitochondrial fragmentation occurred in kidney injury induced by renal ischemia-reperfusion and cisplatin, which was ameliorated by a pharmacological inhibitor of Drp1 [83]. It is also verified by one study using intravital multiphoton microscopy in live animals [84]. Of interest, it is observed that Drp-1-mediated mitochondrial fission also facilitated mitophagy during cisplatin treatment of renal tubular cells [85]. Although it is unclear whether mitochondrial fission can directly trigger mitophagy, fission-associated ROS overproduction and related signals may at least work together to induce mitophagy [86].

Mitochondrial biogenesis and mitophagy coordinate with each other to maintain homeostasis [87]. The mitochondrial life cycle starts with the growth and division of pre-existing organelles (biogenesis) and ends with the degradation of impaired or surplus organelles by mitophagy (turnover). Two crucial transcription factors are responsible for mitochondrial biogenesis, transcription factor A for mitochondria (TFAM) and the peroxisome proliferator-activated receptor-gamma coactivator 1-alpha (PGC1α). Ablation of PGC1α from renal tubule cells suppressed kidney recovery from septic AKI [88], and PGC1α is also pivotal for kidney recovery from ischemic injury by regulating nicotinamide adenine dinucleotide (NAD) biosynthesis [89]. Of interest, decreased TFAM protein level and activity of PGC1α and increased mitophagy were reported cisplatin-treated LLC-PK1 cells, which were prevented by antioxidant αM [90]. One study explored the relationship between PGC1α and mitophagy. It showed that PGC1α could prevent mitophagy impairment in the late kidney injury phase, which accelerates mitochondrial turnover by inducing TFEB to protect against cisplatin AKI [91].

Collectively, PGC1α may be a key regulator of mitochondrial biogenesis and mitophagy, and PGC1α is a promising target for reducing cisplatin nephrotoxicity in cancer therapy. These findings reveal the role of mitochondrial biogenesis and mitophagy after kidney injury.

### 3.3. Roles of Autophagy in Cisplatin Nephrotoxicity and Underlying Mechanisms

#### 3.3.1. Protective Role of Autophagy in Acute Cisplatin Nephrotoxicity

Both pharmacological inhibitors and activators of autophagy were used to explore the role of autophagy in cisplatin nephrotoxicity. 3-MA inhibits autophagosome formation from the beginning, leading to decreased formation of LC3II. Bafilomycin prevents the fusion of autophagosome with lysosome, reducing the degradation of LC3II, while CQ blocks the autophagy flux and impairs cargo clearance [92,93]. Although the mechanisms are different, these autophagy inhibitors increase kidney tubular cell death during cisplatin treatment [69,70]. In contrast, activation of autophagy by rapamycin, one mTOR inhibitor, attenuated tubular injury and protected against cisplatin-induced AKI in mice [70,93,94]. Several natural compounds, such as neferine, emodin, ginsenoside Rb3, and oridonin, were also reported to protect against cisplatin-induced kidney injury through activating autophagy [95,96,97,98,99].

Besides pharmacological approaches, the protective role of autophagy in kidney injury was demonstrated by using conditional autophagy gene knockout mouse models [92,100,101]. Specifically, kidney tubule-specific knockout of *Atg7* [92] or *Atg5* [100,101] worsened AKI induced by cisplatin or renal ischemia-reperfusion, as shown by more severe tubular injury and loss of renal function. Consistently, knockdown of *Beclin1* blocked autophagy and increased apoptosis during cisplatin treatment of renal tubular cells [69,70,102]. Taken together, these studies indicate that autophagy is an intrinsic protective mechanism in kidney tubular cells that are activated rapidly in response to acute cisplatin nephrotoxicity for renal protection (Table 1). Other autophagy-related gene deletion models in kidneys should also be studied in the future.

#### 3.3.2. Mechanisms of the Renoprotective Effect of Autophagy

Autophagy exerts its function by clearing protein aggregates and damaged organelles for reuse or recycling to maintain cellular homeostasis. The loss of this clearing function results in more damaged mitochondria and accumulation of abnormal protein aggregates, as shown in cisplatin-treated proximal tubule *Atg7* [92] or *Atg5* [101] knock-out mice. The *Atg7*-knockout mice also showed more DNA damage and p53 activation than wild-type littermates, suggesting that autophagy dysfunction further exacerbates kidney injury signaling.

While autophagy may protect cells via multiple mechanisms, increasing evidence suggests that mitophagy is a critical defense mechanism in AKI. There are two major pathways of mitophagy: the ubiquitin-dependent pathway and the ubiquitin-independent pathway. The ubiquitin-dependent pathway is also called the PINK1/Parkin pathway, where cell stress-associated loss of mitochondrial membrane potential induces the accumulation of PINK1 on the mitochondrial outer membrane (OMM) where it phosphorylates and recruits Parkin, an E3 ligase, leading to the ubiquitination of OMM proteins. The ubiquitinated mitochondria are then recognized by a series of autophagy receptors with Ub-binding domain and LC3-binding regions, leading to encapsulation by autophagosomes and final degradation in autolysosomes (Figure 2). Initially, it is observed that knockdown of *Pink1* or *Parkin* led to decreased mitophagy and more cell death in cisplatin-treated HK2 cells, which was opposite in overexpression cells [104]. Then, our group [94] demonstrated that *Pink1* or *Parkin*-knockout mice suffered more severe AKI following cisplatin treatment, further supporting a protective role of Pink1/Parkin-mediated mitophagy in cisplatin nephrotoxicity. The ubiquitin-independent pathway involves specific mitophagy receptors, such as BNIP3, BNIP3L, and FUNDC1, which can bind to LC3-II and link damaged mitochondria directly to autophagosomes (Figure 2). In AKI models, BNIP3 is induced in renal tubular cells during ischemic kidney injury and cisplatin nephrotoxicity, and BNIP3 deficiency exacerbated AKI, indicating a protective role of BNIP3-mediated mitophagy in AKI [105,106,107]. In line with this, panax notoginseng saponins, a Chinese herb medicine, protected against cisplatin-induced kidney apoptosis through HIF-1α/BNIP3/BCEN-1 pathway [108]. Mitophagy may be induced as an adaptive response rather than a toxic response in the early phase of kidney injury; however, it might be impaired in the late stage, and this impairment could be prevented by PGC1α, which accelerates mitochondrial turnover by inducing TFEB to protect against cisplatin AKI [91]. These findings suggest that autophagy protect against cisplatin-induced AKI, and this protective effect is mainly related to the clearance of damaged mitochondria via mitophagy.

### 3.4. Regulation of Autophagy in Cisplatin AKI

#### 3.4.1. Energy Signaling Pathway

It is well recognized that nutrient or energy deprivation is a core reason for the induction of autophagy, which is mainly mediated by mTOR, AMPK, and nicotinamide adenine dinucleotide (NAD+) metabolism.

mTORC1 suppresses autophagy by phosphorylating ULK1 and ATG13 to inhibit the ULK1–ULK2 complex formation and autophagy initiation. Several notable autophagy regulators may work by modulating mTOR complex 1 (mTORC1). AMPK may phosphorylate ULK1 to induce autophagy directly or phosphorylate mTORC1 to induce autophagy indirectly. Growth factors regulate mTORC1 activity mainly by activating two classical pathways of mTORC1, PI3K/AKT/mTORC1, and Ras/Raf/MEK/ERK/mTORC1 signaling pathways [109]. In cisplatin AKI, several factors may regulate autophagy through mTORC1. For example, p53 may activate autophagy through the inhibition of mTORC1 by AMPK [110] or miR-199a-3p [111], while activation of protein kinase Cδ (PKCδ) in cisplatin AKI inhibits autophagy through the AKT/mTORC1/ULK1 pathway [112]. Cisplatin-induced NQO1 may inhibit autophagy through activation of the AMPK/TSC2/ mTORC1 signaling pathway [73]. In addition, histone deacetylase (HDAC) inhibitors were shown to protect against cisplatin-induced kidney tubular cell injury [113]. More recent work showed that HDAC inhibitors may protect kidney tubular cells by enhancing autophagy at least partially by suppressing mTORC1 [114]. Well-known inhibitors of mTORC1, rapamycin, and everolimus both affect the cisplatin kidney injury and repair. Together, these studies indicate a role of mTORC1 and related signaling in the regulation of autophagy in cisplatin nephrotoxicity.

Importantly, NAD+ metabolism is critical in regulating autophagy, especially in energy deprivation. NAD+ biosynthesis can promote kidney recovery from ischemic injury in mice [89] and impaired NAD+ biosynthesis is associated with an increased risk of AKI in patients [115]. In addition, NAD+ can reduce the susceptibility to AKI in aged mice through sirt1, one NAD+-dependent class III histone deacetylases [116]. Of note, prophylactic NAD+ boosting by nicotinamide riboside significantly increased autophagy in the ischemic kidney [117]. Thus, the regulation between NAD+ and autophagy possibly depends on sirt1. Under energy depletion, sirt1 activated by increased NAD+ levels can deacetylate ATG proteins [118,119] and increase autophagy genes expression via forkhead box protein O1 (FOXO1) and FOXO3a [120], leading to the autophagy activation. Sirt1 may also deacetylate STK11 to stimulate AMPK and interact with tuberin to inhibit mTORC1 [121] to regulate autophagy and response to stress [122].

#### 3.4.2. Other Pathways

In addition to the energy signaling pathway, other pathways also contribute to the regulation of autophagy. For example, active mitogen-activated protein kinase 8 (MAPK8; also known as JNK1) [123] and death-associated protein kinases (DAP kinases) [124] can activate Beclin 1 to promote autophagy. Some epigenetic mechanisms, kinases, and transcriptional factors are also involved in the regulation of the elongation and fusion stages of autophagy. For example, eukaryotic translation initiation factor 2 subunit-α (eIF2α) [125] and NF-κB kinase inhibitor (IKK) [126] can induce autophagy by regulating some ATG genes. Transcription factor EB (encoded by TFEB) [127] and nuclear export of zinc-finger protein with KRAB and SCAN domains 3 (encoded byZKSCAN3) [128] regulate autophagy by transcriptional regulation of autophagy/lysosomes genes. In cisplatin-induced AKI, activation of TFEB-mediated autophagy and attenuation of mitochondrial dysfunction by trehalose might depend on the inhibition of Akt [129].

## 4. Autophagy in AKI–CKD Transition

After AKI, injured tubules act as a driving force to promote the progression of AKI to chronic kidney disease [130,131,132,133]. In the presence of mild to moderate injury, tubular cells may proliferate to replace apoptotic/necrotic cells, while the cells with severe injury may not recover for proliferation and instead they secrete pro-fibrotic growth factors and inflammatory factors, creating a situation for maladaptive repair [130,131,132,133,134]. The number of autophagosomes was increased in the tubular cells upon and after cisplatin and ischemic AKI [135]. Emerging evidence imply the involvement of autophagy in renal tubular injury and repair during AKI progression to CKD.

Increasing evidence suggests that cell-cycle arrest, especially G2/M arrest, in tubular cells is a pivotal event in maladaptive repair and renal fibrosis through the induction of pro-fibrotic cytokines [136]. Whether autophagy is related to cell cycle arrest and the production of pro-fibrotic cytokines is of interest. Li et al. [15] reported that *Atg5* deficiency in proximal tubules promoted cell cycle G2/M arrest and renal fibrosis in UUO mice and AGT II-treated HK2 cells, while *Atg5* overexpression reversed this outcome. However, Bao et al. [137] reported that G2/M cell cycle arrest was blocked by autophagy inhibitor 3-MA in rats after hyperuricemic nephropathy. Mechanistically, mTOR-autophagy spatial coupling compartment (TASCC) formation increased in G2/M-arrested renal tubular cells [138], and TASCC disruption reduced production and secretion of pro-fibrotic factors in tubular cells after severe AKI. This finding suggests that autophagy participates in TASCC formation in G2/M cell to facilitate maladaptive kidney repair after AKI.

In addition to G2/M arrest, cell senescence, a form of permanent cell cycle arrest, may also participate in the development of CKD, including that induced by cisplatin. Senescence not only prevents cell proliferation but also enhances the production of pro-inflammatory cytokines as part of the senescence-associated secretory phenotype (SASP). Baisantry et al. [15] demonstrated that *Atg5* ablation from kidney proximal tubules decreased tubular cell senescence and reduced fibrosis after renal ischemia/reperfusion injury in mice, implying a role of tubular autophagy in senescence and renal fibrosis in post-injury kidneys. More recently, senescent cells in kidneys after repeated low dose cisplatin treatment, which, along with fibrosis, was alleviated by NAC [62] and intermittent Dasatinib + Quercetin treatment [61].

These observations suggest that autophagy might regulate cell cycle arrest and senescence, affecting kidney repair and AKI–CKD transition after kidney injury.

## 5. Autophagy in Chronic Kidney Problems Following Cisplatin Treatment

In addition to AKI, autophagy has also been implicated in the pathogenesis of CKD transition [8,139]. However, very limited information is currently available on the role of autophagy in chronic kidney problems following cisplatin treatment.

### 5.1. Autophagy in Tubulointerstitial Fibrosis

One pathological hallmark in CKD is interstitial fibrosis. Accumulation of autophagosomes was observed in kidney tubules with tubulointerstitial fibrosis in the UUO model [14,140]. Several studies using specific autophagy gene knockout models revealed that autophagy might promote the progression of tubulointerstitial fibrosis. Compared to wild-type mice, proximal tubule-specific *Atg5* knockout mice had significantly less tubular senescence, less interstitial fibrosis, and improved renal function after renal ischemia/reperfusion injury [15,16]. We [14] demonstrated that mice with *Atg7* knockout in proximal tubules had fewer atrophic tubules, less apoptosis, and less interstitial fibrosis during UUO. These initial findings are supported by follow-up studies [141,142,143], indicating that autophagy in kidney tubules promotes interstitial fibrosis. Mechanistically, autophagy may induce tubular cell death, cell cycle arrest, and senescence, resulting in the production and secretion of pro-fibrotic and pro-inflammatory cytokines to stimulate fibroblasts for fibrosis [14,138]. In particular, Canaud et al. [138] demonstrated the formation of the mTOR-autophagy spatial coupling compartment (TASCC) in renal tubular cells that facilitates the production and secretion of pro-fibrotic factors.

In contrast, there are studies showing evidence for an anti-fibrotic role of autophagy in renal disease models. Global *LC3B (Atg8)* knockout mice and *beclin-1 (Atg6)* heterozygous mice exhibited a deficit in autophagy activation and developed more severe interstitial fibrosis during UUO [17]. Autophagy may suppress fibrosis by promoting the degradation of extracellular matrix proteins including collagens and fibronectin, key components of fibrosis. To support this, Kim et al. [144] showed that inhibition of autophagy increased collagen deposition in TGF β1-treated glomerular mesangial cells (MMC) and in mice during UUO. Additionally, fibronectins, the glycoproteins that connect cells with collagen fibers in the ECM, can also be degraded through autophagy in renal tubular cells [145]. Insight from tubule, *Atg5* knockout proximal tubules showed more severe cell cycle arrest with increased interstitial fibrosis during UUO [15], which implies that its anti-fibrotic effect might be due to its function of cell cycle regulation. Moreover, 3-MA treated kidney in the UUO model showed aggravated tubular cell apoptosis and tubulointerstitial fibrosis, which suggests that autophagy might exert its anti-fibrotic effect by suppressing tubular cell injury [146].

Collectively, these findings suggest that persistent activation of autophagy plays a dual role in kidney fibrosis. On one hand, it may suppress fibrosis by promoting the degradation of extracellular matrix proteins and suppress tubular injury. On the other hand, it may promote fibrosis by inducing a secretory phenotype in tubular cells for the release of pro-fibrotic factors. Currently, autophagy in kidney repair after cisplatin nephrotoxicity and its progression into CKD has not been investigated. Obviously, research in this area may shed light on clinical treatment for cancer patients after cisplatin chemotherapy.

### 5.2. Autophagy and Chronic Inflammation

As mentioned above, chronic inflammation is a critical factor in the pathogenesis of CKD. In 2015, Brooks et al. [147] demonstrated that KIM-1-mediated phagocytosis led to the activation of autophagy to clear apoptotic cell debris and further induced pro-tolerogenic antigen presentation following renal ischemia-reperfusion injury. This study provided the first evidence for autophagy in downregulation of the inflammatory response and immune tolerance maintenance after kidney injury.

Subsequent studies further proved the mutual regulation between autophagy and chronic inflammation in CKD. Inhibition of NLRP3 inflammasome resulted in the upregulation of HIF1α and BNIP3-mediated mitophagy, attenuating apoptosis in contrast-induced AKI [148]. Moreover, NLRP3 knockout in kidney tubular cells showed better renal function and up-regulated mitophagy in UUO, providing evidence for the anti-autophagic function of NLRP3 in kidneys [149]. Reciprocally, prohibitin 2 (PHB2), a newly identified intracellular receptor of mitophagy, protected against kidney tubular injury by improving mitochondrial function and inhibiting NLRP3-induced inflammatory pathways induced by angiotensin II [150]. Moreover, ATG5-mediated autophagy inhibited NF-κB signaling, which alleviated tubular cell inflammation in response to the kidney injury induced by UUO or angiotensin II [151].

To sum up, these findings imply a possible reciprocal regulation between autophagy/mitophagy and chronic inflammation in CKD progression, although it remains unclear if their regulation is direct or indirect.

## 6. Strategies Targeting Autophagy in Cisplatin Nephrotoxicity during Chemotherapy

**Figure 3:**
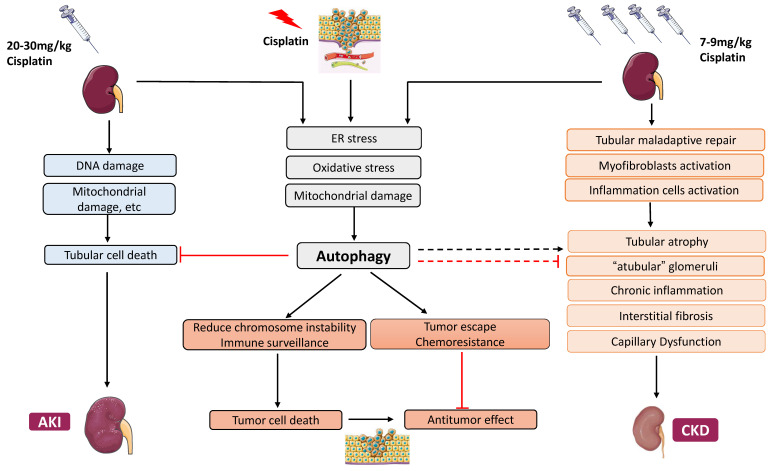
Autophagy in cisplatin-induced nephrotoxicity during cancer therapy. In kidneys, autophagy protects tubular cells against acute kidney injury induced by cisplatin. Autophagy may also affect the development of CKD through its regulation of tubular atrophy, interstitial fibrosis, etc. In cancers, autophagy may suppress tumorigenesis, but it increases the resistance of cancer cells to cisplatin chemotherapy.

Given the critical role of autophagy in cisplatin-induced kidney disease, the pharmacological intervention of autophagy should be a meaningful strategy for the prevention and treatment of kidney diseases during cisplatin chemotherapy. In tumors, autophagy has long been considered a double-edged sword in tumorigenesis and cancer therapy. In the kidney, autophagy is protective during acute cisplatin nephrotoxicity, but the role of autophagy in chronic kidney problems following cisplatin chemotherapy is unclear. Any strategies targeting autophagy in cisplatin chemotherapy must be taken into consideration of the effects in both tumors and normal tissues, especially the kidneys (Figure 3).

### 6.1. Activation of Autophagy

Most pharmacological enhancement of autophagy showed a protective role in the cisplatin-induced kidney injury but makes tumors more sensitive through inhibition of mTOR signaling. However, those drugs might regulate cellular processes beyond autophagy and result in some other effects. For example, mTOR inhibitors, including rapamycin and its analogs everolimus, are widely used autophagy activators. In kidneys, they protect against injury through autophagy but delayed tubular repair following acute injury due to its inhibition of growth and proliferation [152,153]. Additionally, their anti-cancer effects have been well-documented as they prevent tumor cell proliferation and reverse cell senescence [154,155,156] (Table 2). The interventions about upstream mTOR signaling pathways were also conducted. For example, metformin, commonly prescribed for blood glucose control in diabetes, is one with such properties. In kidneys, metformin protected against kidney injury in NRK-52E through stimulating AMPK phosphorylation and autophagy activation [157]. It lowered cancer risks [158] and enhanced the anti-cancer effects of cisplatin on meningioma cells [159] by activating autophagy through AMPK-mTOR pathways and subsequent series of metabolic responses. Trichostatin A and SAHA (broad-spectrum HDAC inhibitors) and Tubastatin A (HDAC6 specific inhibitor) also protects against cisplatin nephrotoxicity in kidneys through AMPK-mTOR pathways but sensitizes cancer cells to cisplatin cytotoxicity by enhancing DNA damage [113,114,160,161,162]. In addition, some natural products, neferine [95,163], emodin [164,165], ginsenoside Rb3 [97], oridonin [98], 3DC2ME [166], scutellarin [167,168], and rottlerin [49,112] also exert the same effect through AKT/ mTOR pathways.

Of interest, trehalose regulates autophagy by rapid and transient lysosomal enlargement and membrane permeabilization (LMP) through TFEB, but does not induce mTOR [129,169]. Although it still benefited the kidney, it also decreased anti-cancer outcomes. It is worth investigating why autophagy caused by mTOR makes the tumor more sensitive, but autophagy induced by TFEB does not show this. There may be two reasons for this situation. The first is that activation of mTOR regulates the upstream of autophagy to cause tumor more sensitive, while TFEB regulates the downstream lysosomal fusion to promote tumor progression. In this case, the interventions stage of autophagy process is particularly pivotal in tumor treatment. The second is that the tumor-killing effect caused by these drugs may be caused by other effects such as DNA damage and inhibition of cell proliferation but not through autophagy.

Besides the above, autophagy activation in some tumors helps the recognition of cancer cells by the immune system to facilitate the anti-tumor immune surveillance and promote autophagic cell death through various pathways of cell death, including apoptosis, necrosis, and necroptosis [170,171]. These effects imply that autophagy might be helpful for cancer prevention and treatment through reducing chromosome instability, increasing immune surveillance, and promoting cell death (Figure 3).

Collectively, although a lot of evidence has shown that drugs that activate autophagy can protect the kidneys and kill tumors, their other pathological effects and whether they play a therapeutic role directly due to autophagy still require in-depth research.

### 6.2. Inhibition of Autophagy

Before tumorigenesis, autophagy defects result in poor elimination of oncogenic proteins, toxic aggregates, and damaged organelles, leading to chronic inflammation, tissue damage, genome instability, and oncogenic gene activation [187,188]. However, autophagy also supports metabolism and survival in cancer cells once the tumor has formed [185]. In this case, autophagy protects cancer cells and increases their resistance to anticancer drugs, and inhibition of autophagy would sensitize cancer cells to therapy and enhance the therapeutic effect of chemotherapeutics.

Multiple studies have demonstrated the evidence for the role of autophagy in cisplatin resistance in chemotherapy. Most animal studies and clinical trials focused on chloroquine (CQ), which blocked autophagic flux and sensitized cancers to cisplatin treatment [18,175,178,179,180,181,182,183,184,185]. Bafilomycin A1, which inhibits autophagy by attenuating the fusion of autophagosome with lysosome, sensitized human bladder cancer cells to cisplatin toxicity [21]. The PI3K pathway is abnormally active in many types of cancers, and PI3K inhibitors block autophagosome formation, thus making PI3K inhibitors attractive targets for cancer therapy through suppressing tumor growth while inhibiting autophagy from sensitizing cancer cells to cisplatin treatment. 3-MA, a P3K inhibitor, enhanced apoptosis and reduced cell viability in cancers [26,174,175,189]. Platinum-resistant ovarian cancer was more sensitive to cisplatin when co-administrated with wortmannin (another PI3K inhibitor) [176], although whether this synergistic effect is due to autophagy inhibition by wortmannin was not determined.

From the current findings, many drugs that inhibit autophagy can decrease the resistance of tumors to cisplatin but can cause damage to acute kidney injury. The function of autophagy in chronic kidney problems following cisplatin treatment remains unclear, but there is evidence for the involvement of autophagy in the development of CKD after kidney injury. In this regard, although inhibiting autophagy aggravates acute kidney injury, it may protect the progression of chronic kidney injury. Therefore, it carefully avoids using autophagy inhibitors in patients with tumors and acute kidney injury, but it may be a promising option for patients with chronic kidney disease. Therefore, it is imperative to verify the role of autophagy in chronic kidney disease.

### 6.3. Therapeutic Potential and Challenges

Despite the progress in research, therapies targeting autophagy remain challenging. First, autophagy might have different roles depending on the disease stage. Autophagy is renoprotective during AKI, but sustained autophagy activation after AKI might promote renal fibrosis or maladaptive kidney repair. It would be beneficial to enhance autophagy during the acute phase of injury but to limit autophagy during kidney repair. It is essential to define these disease phases for appropriate intervention. Second, some autophagy-targeting agents might have other or off-target effects. Due to this, it is sometimes difficult to determine whether the effects of drugs are caused via autophagy or other effects. For example, mTOR inhibitors might protect against AKI by activating autophagy, but they may also prevent kidney repair due to inhibition of cell growth and proliferation [153,154,156]. Third, inhibition of autophagy may sensitize tumors to cisplatin chemotherapy, but it blocks a protective mechanism in kidneys. In this regard, it is crucial to have site-specific delivery of drugs for therapeutic efficacy and safety. Tumor-targeted nanoparticles encapsulated with autophagy inhibitors may emerge as a promising tool to minimize nephrotoxicity during cisplatin chemotherapy. Finally, more clinical trials should be conducted to test, verify and translate those meaningful findings to humans.

## 7. Conclusions

In this review, we have summarized and analyzed the current research about the role and regulation of autophagy in cisplatin-induced AKI and CKD during cancer therapy. Autophagy protects against cisplatin-induced AKI, but its role in CKD development following cisplatin chemotherapy remains unclear. In the future, studies based on tumor-bearing animal models and clinical trials are necessary to determine the safety and effectiveness of autophagy modulators in the kidney and their anti-tumor effects during cisplatin treatment.

## Figures and Tables

**Table 1:** Effects of ablation of autophagy genes on cisplatin-induced AKI.

Variation	Method	Target	Effect	Underlying Mechanisms	Reference
Autophagy	*Atg5* KO	Mouse proximal tubule	More sensitive	1. More damaged mitochondria and abnormal protein aggregates accumulation 2. More DNA damage and p53 activation	[101]
	*Atg7* KO	Mouse proximal tubule	More sensitive	More activation of p53 and c-Jun N terminal kinase	[92]
	*Beclin1* shRNA	NRK-52E cells	Prevent apoptosis	Suppressed apoptosis	[102]
	*Beclin1* siRNA	RPTCs	Aggregated apoptosis and cell death	Sensitized cells to cisplatin-induced apoptosis	[69,70]
Mitophagy	*Pink1/parkin* KO	Mouse global	More sensitive	Promoted Drp1-mediated mitochondrial fission	[94]
	*Pink1* KO	Rat global	Attenuated cisplatin-induced acute kidney injury	Promoted Drp1-mediated mitochondrial fission	[103]
	*Pink1/parkin* knockdown	HK2 cells	More cell injury	Accelerated cisplatin-induced mitochondrial dysfunction	[104]

NRK-52E line, normal rat kidney epithelial cell line; HK-2, human kidney proximal tubule epithelial cell line; Drp1, dynamin-related protein 1; RPTCs, rat kidney proximal tubule epithelial cell line; *Atg*5, Autophagy related 5 gene; and *Atg7*, Autophagy related 7 gene.

**Table 2:** Therapeutic compounds targeting autophagy during cisplatin chemotherapy.

Compound	Kidney			Cancer			
	Model	Effects	Underlying Mechanism	Model	Effects	Underlying Mechanism	Reference
Activate autophagy							
Metformin	NRK-52E	Protective	Stimulate AMPKα phosphorylation	Meningioma cell	Lower cancer risk	AMPK-mTOR pathways	[157,158,159]
Rapamycin	C57BL/6 mice	Protective Harmful	Inhibition of mTOR	Breast cancer Skin tumors	Inhibit progression	1. Inhibition of mTOR 2. Decreased proliferation	[152,155,172]
Everolimus	Wistar/ST rats HK-2 NRK-52E	harmful	Inhibition of mTOR to activates Ulk1 impairing tubular regeneration after acute injury	Ovarian cancer Patients with Thymoma and Thymic Carcinoma	1. Delay development 2. Durable disease control	1. Inhibition of mTOR 2. Reverse cell senescence	[153,154,156]
Trichostatin A (TSA)	C57BL/6mice *Atg7*-/- mice	Protective	AMPK activation and marginal inactivation of mTOR	A2780RES cells	More sensitive	Akt/mTOR Signaling	[114,160]
Tubastatin A (TA)	C57BL/6 HK2 cell	Protective	HDAC6 inhibition decreased renal oxidative stress and malondialdehyde levels	A549 and H292 cells	More sensitive	Increased DNA damage	[114,160]
Rottlerin	PT-*Atg7*-KO mice RPTCs	Protective	Suppress phosphorylation of AKT/ mTOR, p70S6 kinase, and ULK1	A2780 human ovarian cancer cells	More sensitive	Inhibit proliferation, migration, and metastasis	[49,112]
Neferine	NRK-52E cell	Protective	AMPK-mTOR pathways	Human lung adenocarcinoma (A549 cells)	More sensitive	AMPK-mTOR pathways	[95,163]
Emodin	NRK-52E	Protective	Induce the phosphorylation and activation of AMPK, decrease mTOR activation	Human bladder cancer cells Non-small cell lung cancer (NSCLC)	More sensitive	ROS elevation and MRP1 Down-regulation of ERCC1 and inactivation of ERK1/2	[164,165]
Ginsenoside Rb3	HEK293 cell ICR mice	Protective	Regulation of AMPK/mTOR	Oral Cancer	More sensitive	Regulation of AMPK/mTOR	[97]
Oridonin	C57BL/6 mice inhibit A549 cell line activate	Protective	AMPK/Akt/mTOR-dependent autophagosome accumulation	Human lung carcinoma cell	More sensitive	AMPK/Akt/mTOR-dependent autophagosome accumulation	[98]
3DC2ME	LLC-PK1 cells	Protective	AMPK/mTOR	-	-	-	[166]
Scutellarin	C57BL/6 mice	Protective	JNK, p38, ERK and Stat3.	Non-small Cell Lung Cancer	More sensitive	Activate ERK/p53 and c-met/AKT/mTOR signaling pathways	[167,168]
Trehalose	C57BL/6 mice HK2 cell	Protective	1. Activate TFEB-mediated mitophagy 2. Attenuate mitochondrial dysfunction	Me21 cells	Protective	Activate mTOR-independent autophagy	[129,169]
Oleanolic acid	BALB/CN mice Hela cells	Protective	Suppress oxidative stress and inflammatory response	Hela cells hepatocellular carcinoma	More sensitive	Induce autophagic cell death via mTOR	[171,173]
Inhibit autophagy							
3-MA	LLC-PK1 cells RPTC cells	Harmful	Block autophagosome formation-PI3K inhibitors	Osteosarcoma cells Cervical cancer cells Osteosarcoma Human ovarian cancer	More sensitive	Enhanced apoptosis, reduced cell viability	[22,26,70,174,175]
Wortmannin	LLC-PK1 cells	Harmful	Block autophagosome formation-PI3K inhibitors	Platinum resistant ovarian cancer (PROC)	More sensitive	1. Inhibit DNA repair 2. Enhance cellular CP uptake	[176]
LY294002	LLC-PK1 cells	Harmful	Block autophagosome formation-PI3K inhibitors	HCT 166 human colon cancer cell	More sensitive	p53 induction by DNA damage	[177]
Bafilomycin (BAF)	RPTC cells	Harmful	Block fusion of the autophagosome with lysosome	Human bladder cancer	More sensitive	Enhanced apoptosis, reduced cell viability	[70,178]
Chloroquine (CQ)	C57BL/6 mice Adult Wistar rats	Harmful	Block autophagic flux	Human bladder cancer Endometrial cancer Osteosarcoma Nasopharyngeal carcinoma Human gastric cancer Cervical cancer cells	More sensitive	1. Block autophagic flux 2. Autophagy- independent manner	[18,175,178,179,180,181,182,183,184,185]
Morin hydrate	HEK-293 Male ICR mice	Protective	Phosphorylation and activation of AMPK	Hepatocellular Carcinoma	More sensitive	1. Impairing PARP1/HMGB1-dependent autophagy 2. Reverses Cisplatin Resistance	[99,186]

HEK293, human embryonic kidney epithelial cell; LLC-PK1, epithelial-like pig kidney cell line; A549 cell line, Human lung carcinoma cell line; Hela cells, human cervical cancer HeLa cells; A2780RES, cisplatin-resistant human ovarian cancer cells; T or TC, advanced/recurrent thymoma (T) or thymic carcinoma (TC); and Me21, melanoma cells.
